# Reversible Conjugation of Non-ionic Detergent Micelles Promotes Partitioning of Membrane Proteins under Non-denaturing Conditions

**DOI:** 10.1021/acs.langmuir.1c03343

**Published:** 2022-02-18

**Authors:** Mitra Lal, Ellen Wachtel, Mordechai Sheves, Guy Patchornik

**Affiliations:** † The Department of Chemical Sciences, Ariel University, 70400 Ariel, Israel; ‡ Faculty of Chemistry, Weizmann Institute of Science, 76100 Rehovot, Israel; § Department of Molecular Chemistry and Materials Science, Weizmann Institute of Science, 76100 Rehovot, Israel

## Abstract

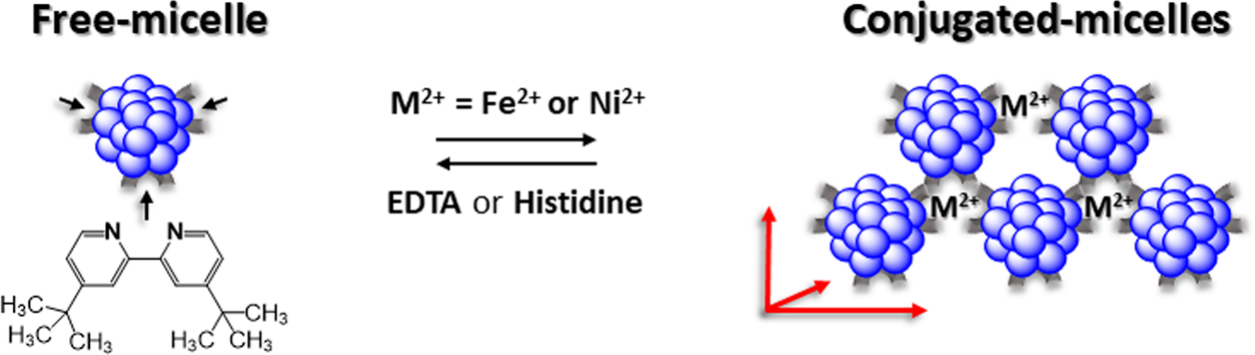

In the decades’-long quest for high-quality membrane protein (MP) crystals, non-ionic detergent micelles have primarily served as a passive shield against protein aggregation in aqueous solution and/or as a conformation stabilizing environment. We have focused on exploiting the physical chemistry of detergent micelles in order to direct intrinsic MP/detergent complexes to assemble via conjugation under ambient conditions, thereby permitting finely tuned control over the micelle cloud point. In the current work, three commercially available amphiphilic, bipyridine chelators in combination with Fe^2+^ or Ni^2+^ were tested for their ability to conjugate non-ionic detergent micelles both in the presence and absence of an encapsulated bacteriorhodopsin molecule. Water-soluble chelators were added, and results were monitored with light microscopy and dynamic light scattering (DLS). [Bipyridine:metal] complexes produced micellar conjugates, which appeared as oil-rich globules (10–200 μm) under a light microscope. DLS analysis demonstrated that micellar conjugation is complete 20 min after the introduction of the amphiphilic complex, and that the conjugation process can be fully or partially reversed with water-soluble chelators. This process of controlled conjugation/deconjugation under nondenaturing conditions provides broader flexibility in the choice of detergent for intrinsic MP purification and conformational flexibility during the crystallization procedure.

## Introduction

Detergent micelles are dynamic structures.^1−5^ Whereas dilute aqueous micellar suspensions may be regarded as ideal solutions in which micelles do not interact,^6^ physical and/or chemical changes in the micellar environment can promote their interactions: (a) inclusion of polymeric precipitants; (b) increase in ionic strength; or (c) change in temperature.^1,6^ Initially isotropic and transparent solutions become turbid, that is, the cloud point is reached, beyond which phase separation into a detergent-rich phase and a detergent-poor phase occurs.^1,6,7^ Such phase separation has been exploited for intrinsic membrane protein (MP) purification.^8−10^ MPs partition spontaneously into the detergent-rich phase, whereas more hydrophilic proteins are rejected.^8,9^ Thus, partial purification of MPs from water-soluble and/or more polar proteins is achieved, and the contaminating background present in MP preparations can be removed. Protein purification relies on detergents capable of reaching cloud point conditions at temperatures that preserve the native conformation of the target MP. To date, this has been achieved primarily with the non-ionic surfactant Triton X-114 (cloud point 22–23 °C).^8−10^ Similar considerations direct MP crystallization protocols.^11^

**Figure 1:**
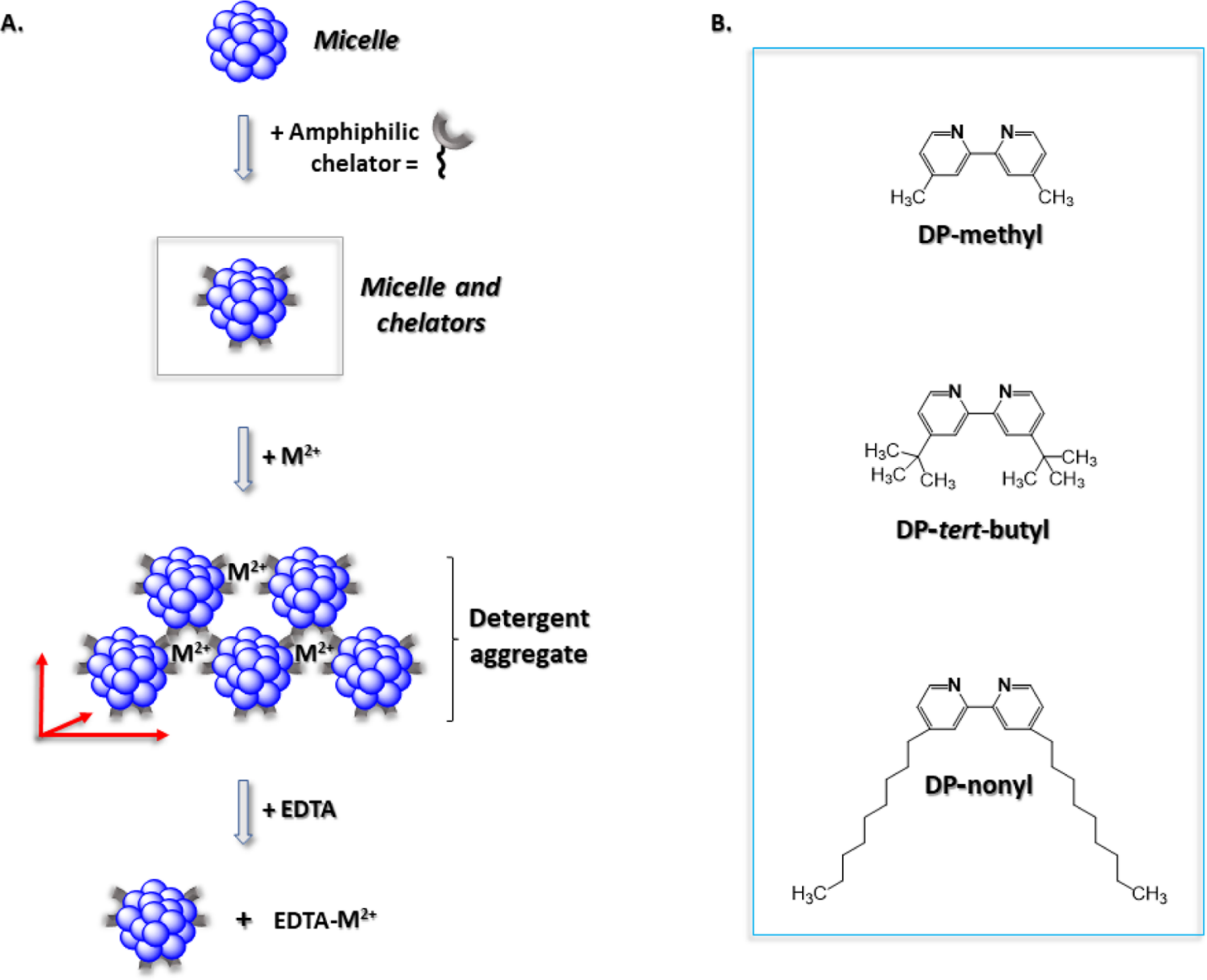
(A) Cartoon of micelle conjugation via amphiphilic metal:chelator complexes generated at the micelle/water interface. Upon addition of EDTA, a strong, water-soluble chelator, the micelle aggregates disassociate. (B) Chemical structures of amphiphilic dipyridine (DP) chelators.

In order to increase the number of detergents beyond those already in use for MP purification, we have developed^12,13^ a micelle conjugation mechanism, which efficiently promotes phase separation at or near room temperature, irrespective of the detergent cloud point (Figure 1A). Here, we explore the behavior of amphiphilic chelators belonging to the bipyridine family: [4,4′-dinonyl-2,2′-dipyridyl (DP-nonyl), 4,4′-di-*tert*-butyl-2,2′-dipyridyl (DP-*tert*-butyl), and 4,4′-dimethyl-2,2′-dipyridyl (DP-methyl)] (Figure 1B). Since bipyridines are known to bind metal ions with lower affinity^14^ than the previously described chelators—bathophenanthroline or phenanthroline derivatives,^15^ the current report raises the possibility of reversing micellar conjugation, either partially or completely, by competition with water-soluble chelators, histidine or ethylenediaminetetraacetic acid (EDTA), respectively. The advantages of such conjugation reversal are twofold: (1) having removed the contaminating background of hydrophilic proteins, and (2) the purified MP/detergent complexes experience additional structural flexibility that could be expected to aid in the growth of well-ordered MP crystal nuclei.

## Materials and Methods

Decyl β-d-maltoside (DM), octyl β-d-glucopyranoside (OG), octyl β-d-1-thioglucopyranoside (OTG), tetraethylene glycol monooctyl ether (C8E4), EDTA, l-histidine, NaCl, FeSO_4_, and NiCl_2_ were obtained from Sigma-Aldrich (St. Louis, MO).

### Conjugation of OG, OTG, C8E4, and DM Micelles Using the [(DP-nonyl)_3_:Fe^2+^] Red Complex

Micellar conjugation required mixing equal volumes of solution A and solution B. Solution A was prepared by the addition of 3 μL of 15 mM DP-nonyl (in EtOH) into 6.25 μL of 200 mM OG [in double distilled water (DDW)] with vigorous vortexing for 20 s. DDW (16 μL) was then added to reach a final volume of 25 μL. Equal volumes of Solution A and Solution B (20 mM FeSO_4_ in DDW) were vortexed for 30 s. Aliquots (e.g., 4 μL) were transferred to siliconized coverslides and incubated at 19 °C over a reservoir (0.5 mL) containing 1 M NaCl in VDX crystallization plates (from Hampton Research). When OTG, C8E4, or DM micelles were studied, 3 μL of 15 mM DP-nonyl was added into 10 μL of either: 100 mM OTG, 100 mM C8E4, or 200 mM DM (all in DDW) with vigorous vortexing for 20 s, and DDW (12 μL) was added to reach a final volume of 25 μL. Here as well, equal volumes of Solutions A and B were mixed, and 4 μL of aliquots was placed for analysis as mentioned above.

### Conjugation of OG, OTG, C8E4, and DM Micelles Using the [(DP-*tert*-butyl)_3_:Fe^2+^] Red Complex

The protocol used here was identical to that of the [(DP-nonyl)_3_:Fe^2+^] complex except for the addition of 1 μL of 130 mM DP-*tert*-butyl (in EtOH) into 6.25 μL of either 200 mM OG or into 10 μL of 100 mM OTG, 100 mM C8E4, or 200 mM DM (all in DDW) and further addition of DDW to a final volume of 25 μL after vigorous vortexing.

### Conjugation of OG, OTG, C8E4, and DM Micelles Using the [(DP-methyl)_3_:Fe^2+^] Red Complex

The protocol used here was the same as described for the [(DP-nonyl)_3_:Fe^2+^] complex except for the addition of 3 μL of 15 mM DP-methyl (in EtOH) into 6.25 μL of either 200 mM OG or into 10 μL of 100 mM OTG, 100 mM C8E4, or 200 mM DM (all in DDW) and addition of DDW to a final volume of 25 μL after vigorous vortexing.

### Conjugation of OG, OTG, C8E4, and DM Micelles Using the [(Bipyridine)_3_:Ni^2+^] Complexes

Conjugation was performed via the same protocols as described above except for replacing the 20 mM FeSO_4_ solution with 20 mM NiCl_2_ solution (both in DDW).

### Conjugation of Bacteriorhodopsin (bR) in OTG Micelles Using the [(DP-nonyl-butyl)_3_:Ni^2+^], [(DP-*tert*-butyl)_3_:Ni^2+^], or the [(DP-methyl)_3_:Ni^2+^] Amphiphilic Complexes

Cells of *Halobacterium salinarum* were grown, as previously described,^16^ and bR purple membrane fragments were isolated from washed cells, according to an established procedure.^17^ bR is a small integral MP (26 kDa) containing a retinal chromophore covalently bound via a protonated Schiff base to Lys216. The protein utilizes light energy for the active transport of protons from the cytosol to the extracellular side of the membrane and uses the generated proton gradient for the synthesis of ATP by ATP synthase. bR was extracted from purple membranes with OTG, as described previously.^18^ To freshly extracted bR (10.5 μL of 3–5 mg/mL bR), 0.5 μL of 27 mM DP-nonyl or DP-*tert*-butyl or DP-methyl (in EtOH) was added with vigorous stirring (for 20 s) and followed by the addition of 2.5 μL of 5.4 mM NiCl_2_ (in DDW). Aliquots (4 μL) from the latter mixture were transferred to siliconized coverslides and incubated overnight at 19 °C in the dark against a reservoir (0.5 mL) containing 0.5 M NaCl in VDX crystallization plates (from Hampton Research).

### Addition of Water-Soluble Chelators

40 mM EDTA or 40 mM histidine was added to the suspensions of conjugated micelles prepared, as described in the experimental section.

### Dynamic Light Scattering

(I) **Individual micelles**: Samples for dynamic light scattering (DLS) measurements (0.5 mL final volume) were prepared by mixing 375 μL of DDW with 125 μL of 0.2 M OG; or 400 μL of DDW with 100 μL of 0.2 M DM; or 450 μL of DDW with 50 μL of either 0.2 M OTG or 0.2 M C8E4. This was followed by 5 min centrifugation at a relative centrifugal force (RCF) 21,000; and 400 μL of the resulting supernatant was used for determining the hydrodynamic size distribution. (II) **Conjugated micelles**: Micellar conjugation was initiated by adding 24 μL of 15 mM DP-nonyl and 40 μL of 0.1 M FeSO_4_ in DDW to the original 400 μL samples. Following 20 min incubation at 19 °C, DLS measurements were then immediately performed. (III) **Process reversibility with EDTA or histidine**: To 464 μL of conjugated OG, OTG, C8E4, or DM micelles, 80 μL of 0.2 M EDTA (at pH 7.5) or 80 μL of 0.2 M histidine (at pH 7.5) was added and incubated at 19 °C for different periods of time. This was followed by 10 min centrifugation at a RCF 21,000 using Ultrafree MC-VV centrifugal filter Durapore [poly(vinylidene difluoride), 0.1 μm] prior to analysis. Intensity-weighted size distributions were determined using the auto correlation spectroscopy protocol of a NANOPHOX instrument (Sympatec GmbH, Germany).

### Light Microscopy

Images were obtained using an Olympus CX-40 light microscope equipped with an Olympus U-TV1X-2 digital camera.

## Results and Discussion

**Figure 2:**
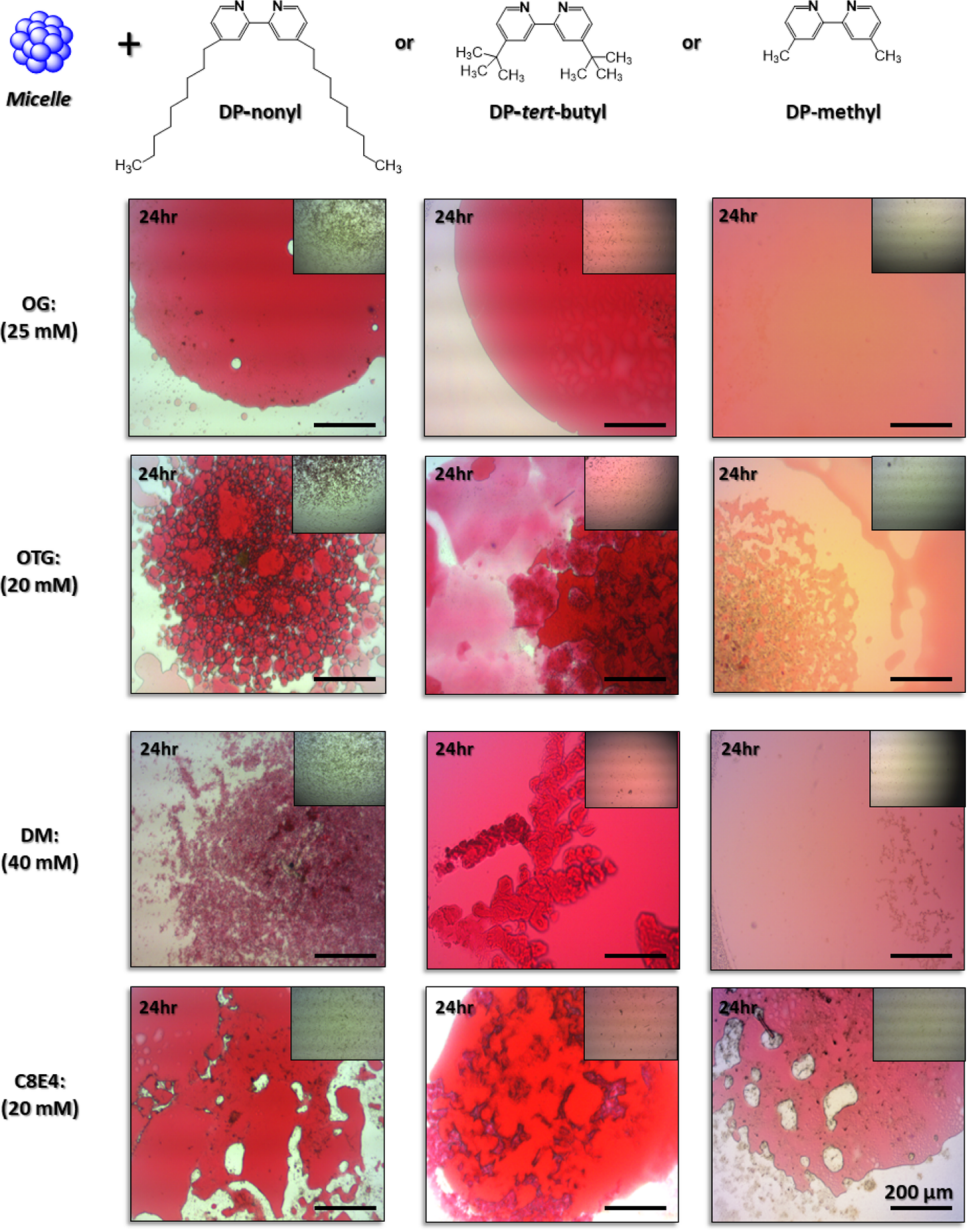
Light microscopy images taken 24 h after conjugation of octyl glucoside (OG), octylthioglucoside (OTG), decyl maltoside (DM), and tetraethylene glycol monooctyl ether (C8E4) micelles with bipyridine analogue chelators and 10 mM Fe^2+^. Insets show images of the system immediately after addition of Fe^2+^. Scale bar indicates 200 μm.

Three commercially available bipyridine analogues with increasing hydrophobicity were studied (Figure 1B). In the most hydrophobic analogue, DP-nonyl, each of the pyridine rings in the bipyridine moiety is bound to an aliphatic tail comprising nine carbons (Figure 1B), thereby anchoring the chelator in the micelle core. In DP-*tert*-butyl or DP-methyl, each pyridine ring is bound to four or one carbon atoms, respectively, and hence, these chelators are less lipophilic (Figure 1B). Addition of DP-nonyl to a non-ionic detergent micellar suspension (OG, OTG, DM, or C8E4), followed by Fe^2+^ ions, which can bind three bipyridine moieties,^14^ led after 24 h of incubation at 19 °C to phase separation in the form of red, oil-rich globules, ranging in size from 15 to 1000 μm (Figure 2, left hand side). The input (detergent: DP-nonyl) stoichiometric ratios during micellar conjugation was 1:28 for OG, 1:22 for OTG or C8E4, and 1:44 for DM, respectively. Since the aggregation number (*N*_A_) for OG is ∼100,^19^ for OTG (*N*_A_ ∼ 114^20^), for C8E4, (∼95^21^), and DM (∼125^19^), it follows that a maximum ∼3–4 chelators per micelle are required to trigger phase separation.

The color of the globules derives from the [(bipyridine)_3_:Fe^2+^] red complex.^22^ Whereas phase separation was readily observed with all detergents tested in the presence of the DP-nonyl chelator, DP-methyl promoted phase separation only with OTG and C8E4 (Figure 2), and DP-*tert*-butyl promoted phase separation with all detergents except for DM (Figure 2). This finding is consistent with the observed increased hydrophobicity of DP-nonyl as compared to the other two dipyridine (DP) analogues. In order to avoid generation of red color that might interfere with spectroscopic analyses, micelle conjugation with cations other than Fe^2+^ was also tested. We found that replacing Fe^2+^ ions by Ni^2+^ led to clear phase separation with all (chelator:detergent) combinations (Supporting Information, Figure S1). We note that the binding affinity of the bipyridine moiety to Ni^2+^ is 1000-fold higher than that of Fe^2+^.^14^

**Figure 3:**
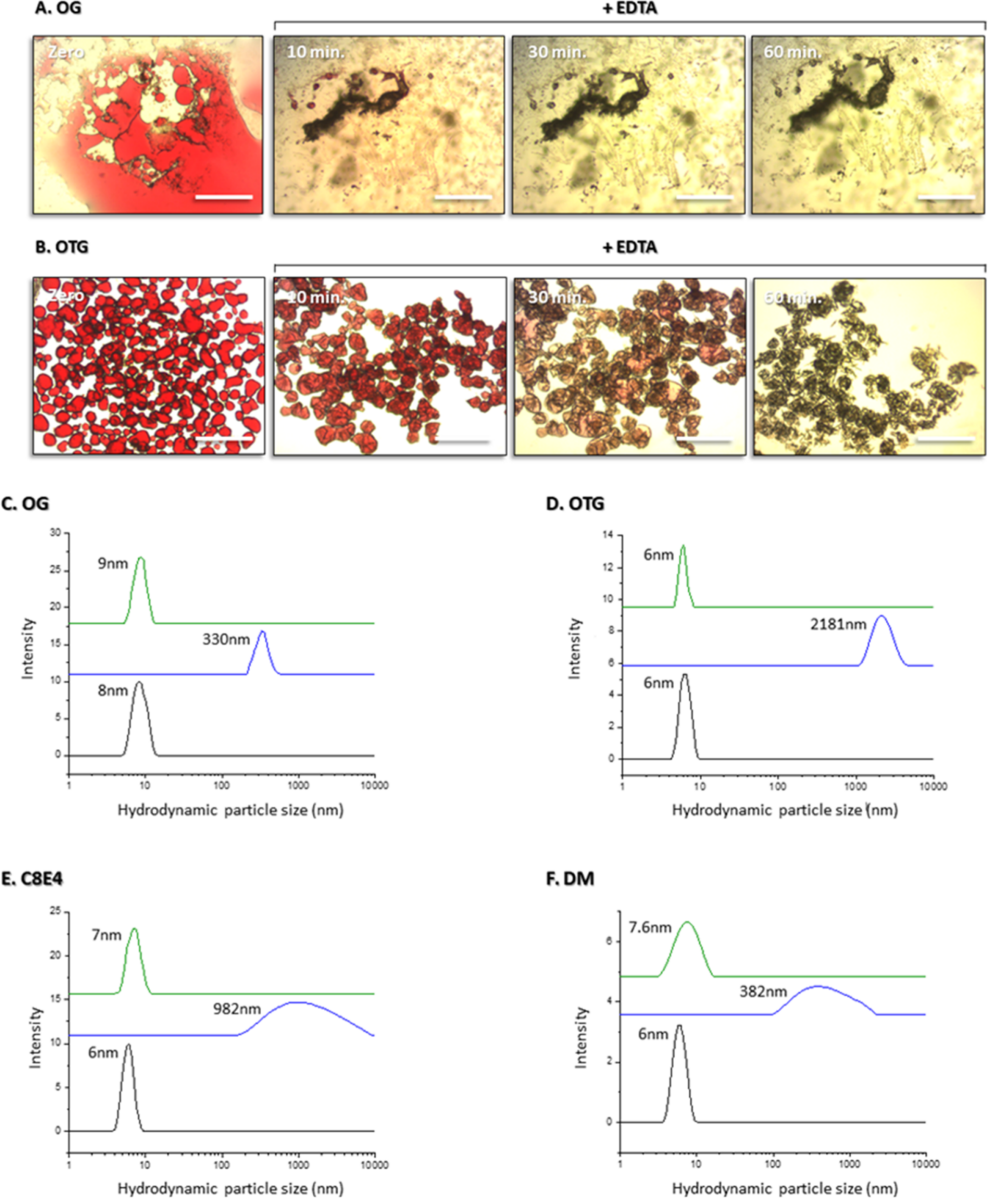
Micelle conjugation reversibility observed after addition of EDTA. (A,B). Light microscope images of red, oil-rich globules generated following the conjugation of detergent micelles with [(DP-nonyl)_3_:Fe^2+^] amphiphilic complexes (for experimental details, see the Materials and Methods section). Following phase separation, addition of 40 mM EDTA results in the disappearance of the red colored, oil-rich globules. Scale bar indicates 200 μm. (C–F). Dynamic light scattering (DLS) analysis of micellar conjugation and its reversal. Black lines—individual detergent micelles; blue lines—following 20 min incubation with the amphiphilic [(DP-nonyl)_3_:Fe^2+^] complex; green lines—following addition of 33 mM EDTA and 1 h incubation. Detergent concentration for DLS analysis: OG—50 mM; OTG—12 mM; C8E4—20 mM; and DM—40 mM.

In an attempt to introduce additional conformational freedom to inter-micellar interactions, we explored the possibility of adding EDTA to compete for metal binding following OG micelle conjugation with the [(DP-nonyl)_3_:Fe^2+^] complex and subsequent oil-rich globule formation (Figure 3A). We observed that 10 min after addition of 40 mM EDTA, the majority of the red globules had disappeared. The rapid kinetics of conjugation reversal would argue that conjugating OG micelles does not produce fusion. Similar behavior was observed with C8E4 and DM micelles (Supporting Information, Figure S2A,B). However, more than 30 min were required to dissociate conjugated OTG micelles and eliminate the red color (Figure 3B). The presence of a sulfur atom bound to the anomeric carbon of glucose in OTG, making this detergent more lipophilic than OG, may be responsible for the slower response.

DLS provided evidence on the submicron scale for reversal of micelle conjugation with the [(DP-nonyl)_3_:Fe^2+^] complex (Figure 3C–F). Consistent with light microscopy images, we found that 20 min after introduction of the amphiphilic complex to the micellar dispersion, individual micelles (6–9 nm) were no longer detectible in the suspension. Rather, particles more than 2 orders of magnitude larger (i.e., hydrodynamic diameter 330–2105 nm) were generated with all detergents tested (Figure 3C–F). Subsequent addition of 40 mM EDTA recovered micelles with hydrodynamic size approximately the same as that measured prior to micelle conjugation (Figure 3C–F). A weaker chelator, histidine, led to the appearance of two particle populations: one that was approximately the same as the original micelle and a second particle that ranged between 382 and 2105 nm (Supporting Information, Figure S3A–D). This observation is consistent with the fact that histidine is less capable of competing for Fe^2+^ ions complexed with the bipyridine moiety.

**Figure 4:**
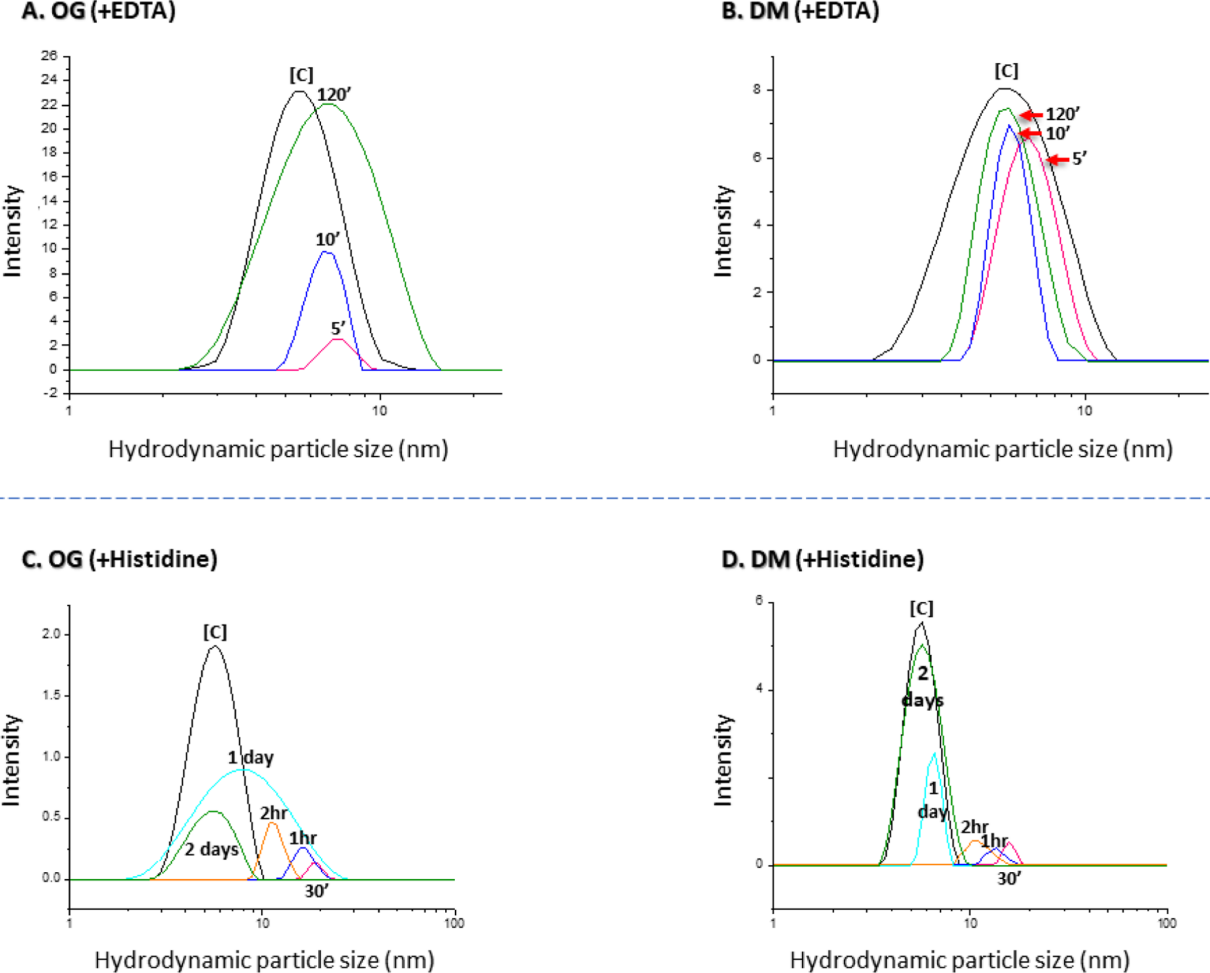
Time course of the recovery of individual OG or DM micelles after addition of either 33 mM EDTA (A,B) or 33 mM histidine (C,D). [C]—Initial OG or DM micellar peak. Detergent concentration for DLS analysis: OG—50 mM; OTG—12 mM; C8E4—20 mM; and DM—40 mM.

Time course experiments with conjugated OG or DM micelles show that 33 mM EDTA can essentially quantitatively reverse the conjugation process within 2 h with both detergents (Figure 4A,B), whereas histidine (at the same concentration) required 2 days of incubation and led to quantitative recovery of only DM but not OG micelles (Figure 4C,D). These findings provide additional support for the assumption that a stronger chelator is more capable of competing with the bipyridine moiety for metal binding and, hence, reverses the process more rapidly and more efficiently.

**Figure 5:**
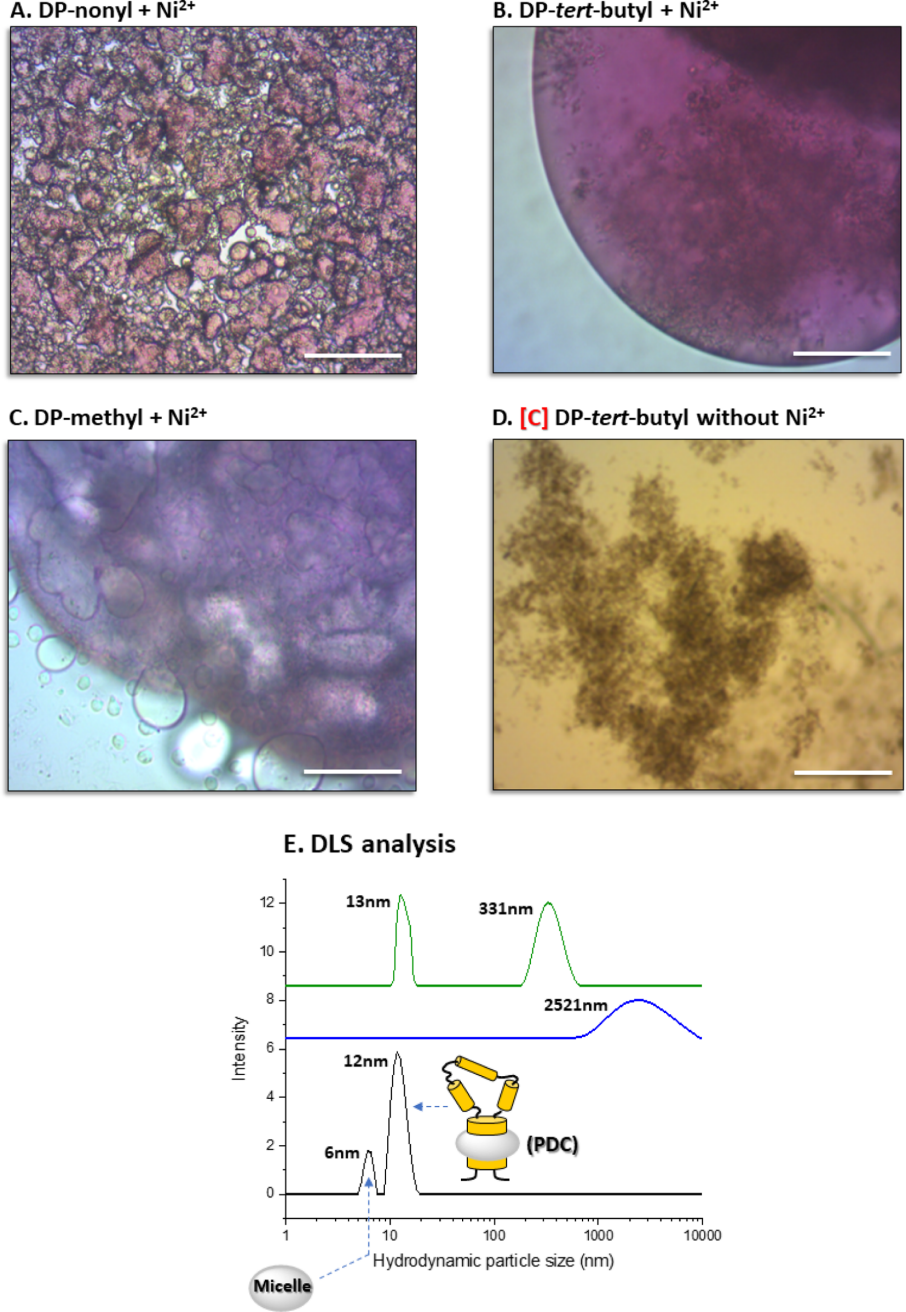
Conjugation, via [(bipyridine)_3_:Ni^2+^] complexes, of PDCs containing the MP (bR) embedded in OTG/phospholipid mixed micelles. (A–C) Light microscopy images showing formation of purple globules generated after conjugating PDCs using 1 mM of [(DP-nonyl)_3_:Ni^2+^], [(DP-*tert*-butyl)_3_:Ni^2+^], or [(DP-methyl)_3_:Ni^2+^] complexes. (D) Control experiments showing the formation of dark precipitate when Ni^2+^ is not added together with DP-*tert*-butyl. Scale bar represents 200 μm. (E) DLS analysis: black line: bR extracted from its native membrane with OTG; blue line: 2.5 h after addition of 1 mM [(DP-*tert*-butyl)_3_:Ni^2+^]; and green line: following addition of 40 mM EDTA.

We further tested the possibility of conjugating protein detergent complexes (PDCs), containing OTG, native phospholipids, and the bacterial, light-driven proton pump, bacteriorhodopsin (bR),^23^ with the three [DO:Ni^2+^] complexes and assessed process reversibility. We found that overnight incubation at 19 °C of bR PDCs with the three amphiphilic complexes led to purple globules (Figure 5A–C). Preservation of the purple color verified the native conformation of the retinal chromophore covalently bound in the protein.^24^ Interestingly, in the presence of DP-nonyl but without Ni^2+^, the protein denatured and lost its purple color (Figure 5D), thereby emphasizing the importance of conjugation for preservation of the MP native state.

DLS measurements demonstrated that the extracted PDC preparation contains OTG micelles devoid of bR (6 nm) as well as those containing bR (12 nm) (Figure 5E, black line). Incubation during 2.5 h at 19 °C following the addition of 1 mM [(DP-*tert*-butyl)_3_:Ni^2+^] amphiphilic complex leads to the disappearance of both the 6 and 12 nm peak, while generating a new peak at 2521 nm (Figure 5E, blue line). This marked difference in the particle size is consistent with effective PDC conjugation. Addition of 40 mM EDTA following PDC conjugation regenerates a peak at 13 nm, as well as at 331 nm, in parallel with the disappearance of the peak at 2521 nm (Figure 5E, green line). These results imply that conjugation of PDCs containing bR can be reversed, at least partially, with EDTA.

It therefore follows that a hydrophobic environment can be generated with amphiphilic [bipyridine:metal] complexes and provide a realistic route for purification of MPs with detergents other than Triton X-114. The ability to conjugate micelles and to generate distinct new phases: (a) independent of the detergent cloud point; (b) regardless of the nature of the detergent headgroup (e.g., glucose, maltose, and ethylene glycol); (c) at 19 °C and perhaps also at lower temperatures; (d) without the need of a precipitant (e.g., ammonium sulfate or poly(ethylene glycol)), presents a simple route toward partial purification of MPs or their concentration as part of a crystallization trial. However, we do note that the higher detergent concentration within the oil-rich globules may denature particularly labile MPs. Therefore, the suitability of the separation/purification approach we have presented should be assessed on a case-by-case basis.

**Figure 6:**
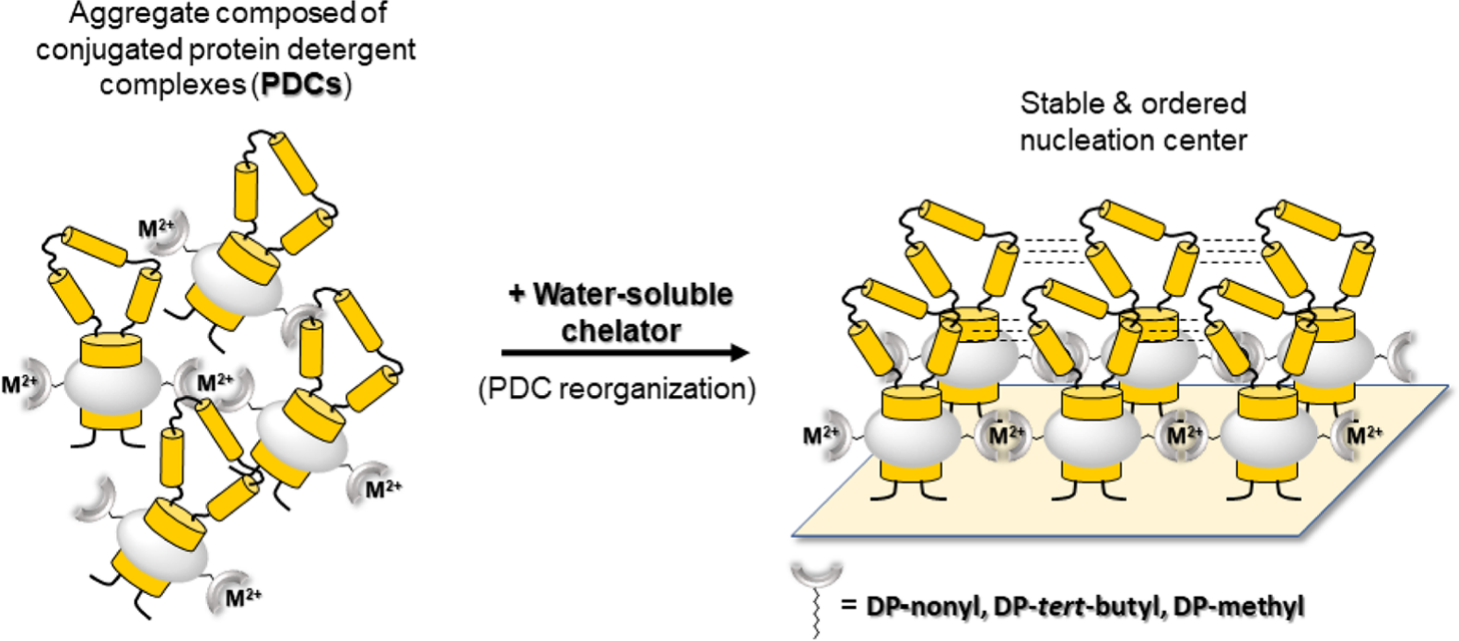
Cartoon showing how PDCs can be brought into proximity with [bipyridine:M^2+^] amphiphilic complexes and undergo dissociation/association events in the presence of a water-soluble chelator. Such “breathing events” between PDCs should allow the latter to reorient themselves until the most stable, organized assembly is reached. Organized clusters of PDCs stabilized by [bipyridine:M^2+^] complexes may represent nucleation centers that would support crystal growth of MPs embedded in micelles.

In addition, the synthetic “cloud point” may be utilized for structure determination of intrinsic MPs as most 3D crystals of this class of proteins are composed of MPs embedded in micelles.^25^ Therefore, amphiphilic [bipyridine:metal] complexes may provide a simple to implement, non-denaturing avenue for bringing PDCs into proximity and at the same time, due to the low binding affinity of the bipyridine moiety to metal cations, allow PDCs to undergo dissociation–association events until an organized and, hence, stable nucleation center is reached to support the crystal growth (Figure 6).

## Conclusions

In summary, amphiphilic [(DP-nonyl)_3_:Fe^2+^], [(DP-*tert*-butyl)_3_:Fe^2+^] complexes, along with their analogous Ni^2+^ derivatives, were found to conjugate non-ionic detergent (OG, OTG, DM, or C8E4) micelles, in a strikingly efficient manner. Micelle conjugation leads to macroscopic phase separation under ambient conditions, independent of the detergent cloud point temperature. Comparing these observations with our earlier studies on phenanthroline analogues, which conjugate micelles irreversibly,^12,26^ we conclude that process reversibility in the presence of competing water-soluble chelators, EDTA or histidine, likely derives from the relatively low binding affinity of the bipyridine moiety for Fe^2+^ and Ni^2+^ ions. Partial or complete reversal of micelle conjugation at temperatures close to ambient via competition with weak (histidine) or strong (EDTA) metal chelators should assist in (a) separating intrinsic MPs in oil-rich globules from contaminating background (hydrophilic) proteins, (b) promoting crystal growth of purified, intrinsic MPs by directing MP-detergent complexes to cluster under non-denaturing conditions while, in parallel, allowing sufficient freedom for nucleation centers to undergo energetically favorable structural rearrangements. With the results presented above as a strong foundation, our future work will be directed toward these goals.

## Supporting Information Available

Additional light microscopy images and DLS measurements of conjugated OG, OTG, DM, and C8E4 micelles (PDF)
