# Supplementary material for: Reversible Conjugation of Non-ionic Detergent Micelles Promotes Partitioning of Membrane Proteins under Non-denaturing Conditions
Source: Langmuir. 2022 Feb 18;38(8):2626–33. doi: 10.1021/acs.langmuir.1c03343 (PMC8892955; doi:10.1021/acs.langmuir.1c03343)
Supplement: Supplementary file 1 — la1c03343_si_001.pdf [file la1c03343_si_001.pdf]

Reversible conjugation of non-ionic detergent micelles promotes partitioning of membrane proteins under non-denaturing conditions

Supplementary Material

Mitra Lal [1], Ellen Wachtel [2], Mordechai Sheves [3] and Guy Patchornik [1*]

[1] Department of Biological Chemistry, Ariel University, 70400, Israel.

[2] Faculty of Chemistry, Weizmann Institute of Science, 76100, Rehovot, Israel.

[3] Department of Molecular Chemistry and Materials Science, Weizmann Institute of Science, Rehovot, 76100, Israel.

*Corresponding author:

Email: guyp@ariel.ac.il

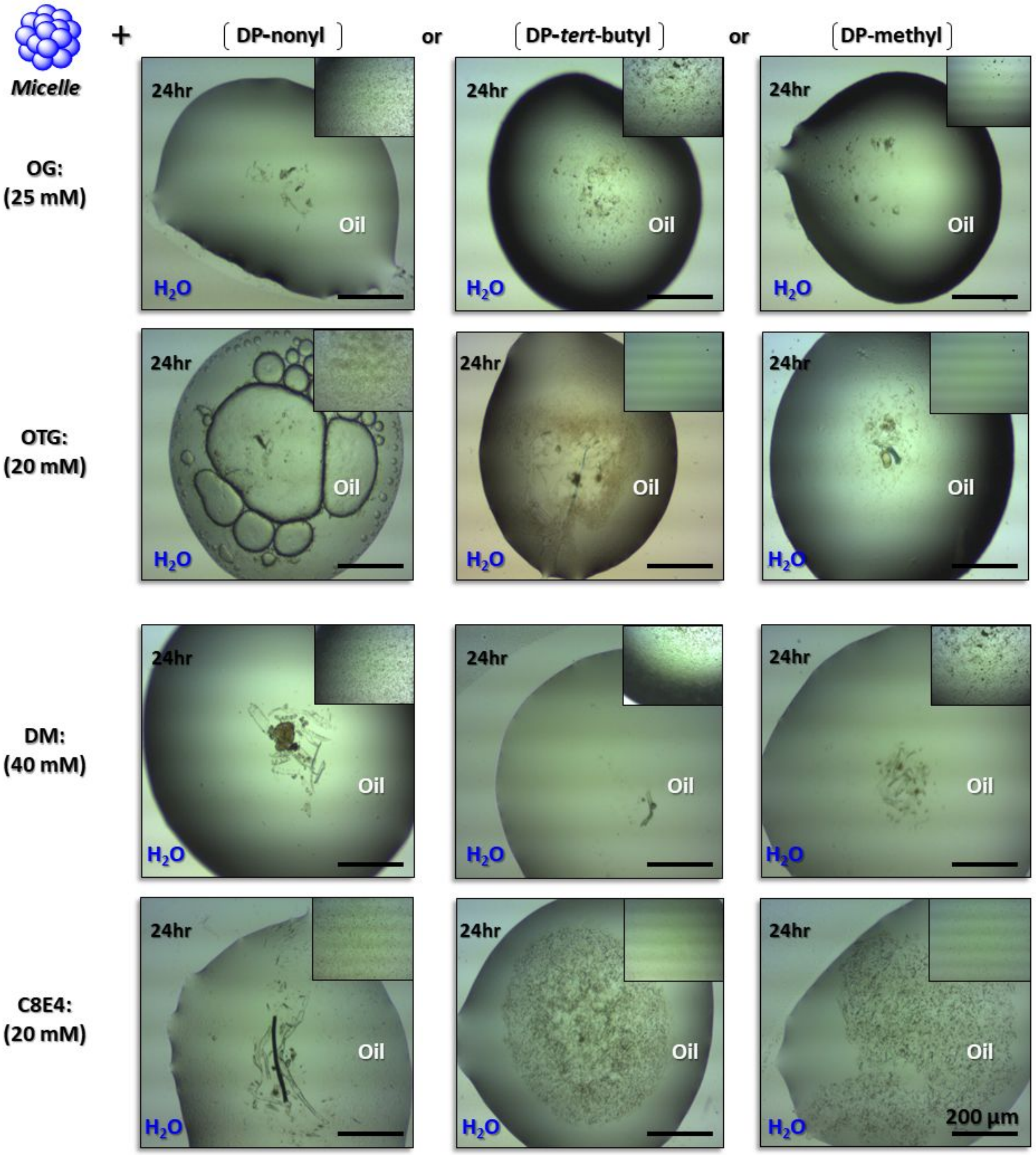

Figure S1: Light microscopy images taken 24 hrs after conjugation of OG, OTG, DM and C8E4 micelles with bipyridine analog chelators and 10 mM Ni^{2+}. Scale bars indicate 200 µm. Insets are images of the system immediately after addition of Ni^{2+}.

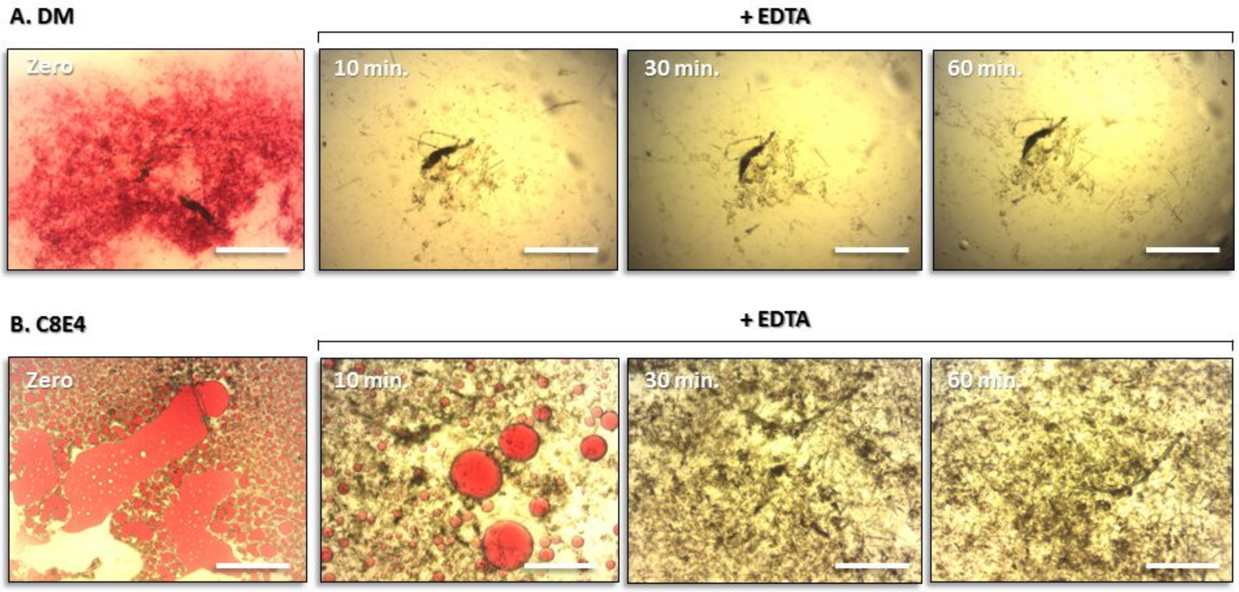

Figure S2: Conjugation reversibility with the water-soluble, strong chelator EDTA following micellar conjugation using [(DP-nonyl)$_3$:Fe^{2+}]. Micelles comprising (A) DM or (B) C8E4 non-ionic detergents were conjugated with the red [(DP-nonyl)$_3$:Fe^{2+}] amphiphilic complex until a distinct oil-rich phase was observed. EDTA (40 mM) was then added and the time dependence of the disappearance of the red color and oil-rich globules was followed at 19°C. Scale bars indicate 200 μm.

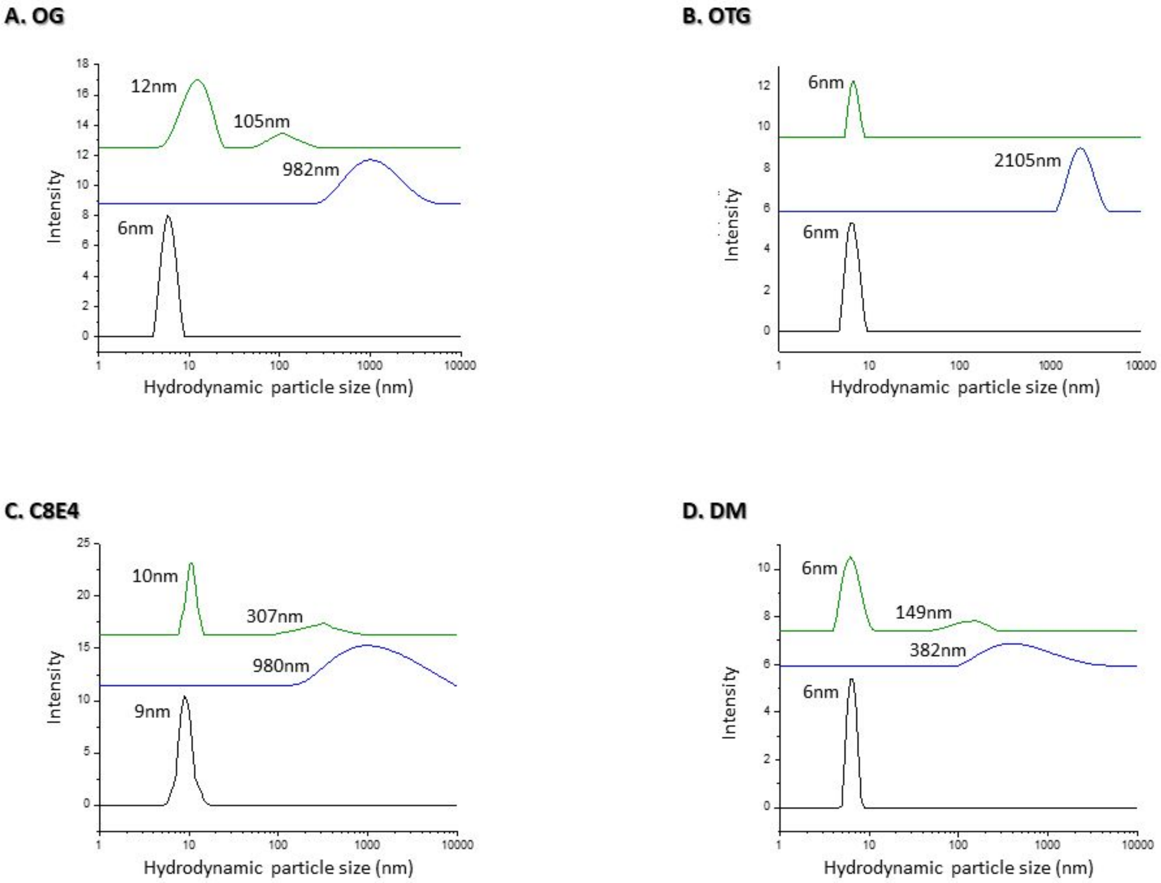

Figure S3: Micelle hydrodynamic size distribution studied with dynamic light scattering (DLS) **A-D.** Black lines - unconjugated micelles; Blue lines - following 20 minutes incubation with the amphiphilic [(DP-nonyl)$_3$:Fe^{2+})] complex; Green lines - following addition of 33 mM histidine and overnight incubation at 19 ^{o}C.
